# Intermediate water circulation drives distribution of Pliocene Oxygen Minimum Zones

**DOI:** 10.1038/s41467-022-35083-x

**Published:** 2023-01-04

**Authors:** Catherine V. Davis, Elizabeth C. Sibert, Peter H. Jacobs, Natalie Burls, Pincelli M. Hull

**Affiliations:** 1 Department of Marine, Earth, and Atmospheric Sciences, North Carolina State University, Raleigh, NC USA; 2 Department of Earth and Planetary Sciences, Yale University, New Haven, CT USA; 3 Yale Institute for Biospheric Studies, Yale University, New Haven, CT USA; 4 Department of Environmental Science and Policy, George Mason University, Fairfax, VA USA; 5 Department of Atmospheric, Ocean & Earth Sciences, George Mason University, Fairfax, VA USA; 6 Yale Peabody Museum of Natural History, Yale University, New Haven, CT USA; 7 Present Address: Earth Science Division, NASA Goddard Space Flight Center, Greenbelt, MD USA

**Keywords:** Marine chemistry, Palaeoceanography

## Abstract

Oxygen minimum zones (OMZs) play a critical role in global biogeochemical cycling and act as barriers to dispersal for marine organisms. OMZs are currently expanding and intensifying with climate change, however past distributions of OMZs are relatively unknown. Here we present evidence for widespread pelagic OMZs during the Pliocene (5.3-2.6 Ma), the most recent epoch with atmospheric CO_2_ analogous to modern (~400-450 ppm). The global distribution of OMZ-affiliated planktic foraminifer, *Globorotaloides hexagonus*, and Earth System and Species Distribution Models show that the Indian Ocean, Eastern Equatorial Pacific, eastern South Pacific, and eastern North Atlantic all supported OMZs in the Pliocene, as today. By contrast, low-oxygen waters were reduced in the North Pacific and expanded in the North Atlantic in the Pliocene. This spatially explicit perspective reveals that a warmer world can support both regionally expanded and contracted OMZs, with intermediate water circulation as a key driver.

## Abstract (web summary)

The Pliocene shows that a warmer world can support both expanded and contracted marine Oxygen Minimum Zones. While oxygen distributions were overall like today, there was less low-oxygen water in the North Pacific and more in the North Atlantic

## Introduction

The presence and distribution of low-oxygen water masses are of global importance to biogeochemical and nutrient cycling and may present a barrier for many marine organisms^1–7^. A link between anthropogenic climate change and marine deoxygenation has become clear over the past decades, with growing evidence for an expansion of oxygen minimum zones (OMZs) and coastal hypoxia^4,6,8–11^. Oxygen minimum zones can be broadly defined as open ocean, mid-waters with oxygen content markedly lower than the surface, usually reaching greatest intensity between 200 and 1000 m depth. Quantitative definitions vary widely between publications. As most paleo-oxygen proxies are not quantitative we converge upon a qualitative definition of an OMZ as a region of subsurface dissolved oxygen levels low enough to effect biological or chemical gradients. Although the extent and intensity of OMZs fluctuate on decadal to millennial time scales^12–16^, the future distribution of marine oxygenation in a warmer climate remains unclear. This is because long-term, secular changes in the distribution of OMZs depend on the complex interplay of multiple factors that include global and local shifts in intermediate water circulation, stratification, export productivity and remineralization, and ocean temperatures, the balance among which is not well understood.

**Fig. 1:**
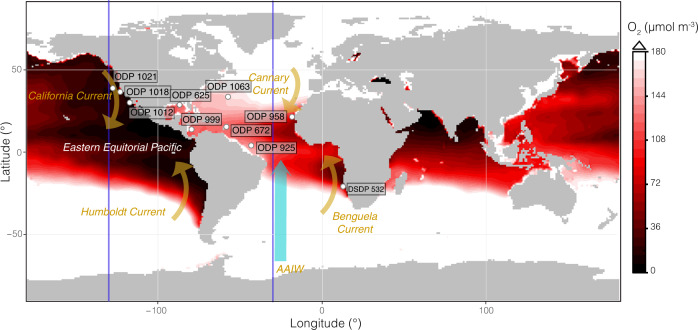
Distribution of low-oxygen mid-waters in the modern ocean. Minimum O_2_ within the upper 1000 m from WOA18 data (1955–2010)^90^ is shown with lower O_2_ concentrations in darker red. Regions where oxygen concentrations do not fall below 172 μmol m^-3^ are white. Dark blue lines show the location of sections expanded in Fig. 5. Key surface currents are shown in orange and Antarctic Intermediate Water (AAIW) in light blue All core sites discussed in the text are shown as white points. A Pliocene comparison is available as Supplementary Fig. 7.

In such complex systems, combining modeling and data-driven approaches offers a powerful tactic for disentangling the relative importance and interactions of potential drivers under different boundary conditions. For instance, modern OMZs are frequently associated with regions where surface productivity is high and ventilation of intermediate source waters is low, typified by Eastern Boundary Upwelling Systems (EBUSs)^17^ (Fig. 1). However, the degree to which OMZs have been retained in, or confined to, these types of environments under conditions of both variable productivity and ventilation has not been widely explored. Here we investigate OMZs during the Pliocene Epoch (5.3-2.6 million years ago; Ma). The mid-Piacenzian, also known as the mid-Pliocene Warm Period (~3 Ma), has received particular interest as the most recent period during which atmospheric CO2 was comparable to present (~400–450 ppm^18–20^), with global mean temperatures ~2–3°C warmer than the 21st century^21–23^. As a result, the Pliocene has been the focus of multiple generations of the Pliocene Research, Interpretation and Synoptic Mapping (PRISM) project and several major data-model studies comparing PRISM reconstructions to Pliocene climate model simulations like the Pliocene Model Intercomparison Project (PlioMIP)^23,24^.

Despite the similarities in *p*CO_2_ and continental configurations between the mid-Piacenzian and modern, there were several key differences in Pliocene oceans that have the potential to impact the extent, intensity, and distribution of OMZs. This includes the presence of distinct ocean circulation patterns, such as a Pacific Meridional Overturning Circulation (PMOC), which has no modern analogue^25,26^ but could affect the distribution and age of intermediate water masses. Some models of Pliocene-like climate suggest globally reduced upwelling-conducive winds due to reduced meridional temperature gradients^27,28^. This could act to reduce nutrient input and export productivity and decrease the strength and extent of OMZs especially in EBUSs. Empirical evidence for this last scenario is mixed, with nitrogen isotopes indicating a reduction in productivity in both the Benguela^29,30^ and California Current systems^31^, but with increased calcium carbonate mass accumulation rates also observed in the California Current^32^. Meanwhile, warmer surface and intermediate waters in the Pliocene would have decreased oxygen solubility, potentially countering the effect of reduced upwelling and promoting expanded OMZs. In addition, while paleogeography by the end of the Piacenzian closely resembled today’s continental configuration, the influence of the closure of the Central American Seaway during the Pliocene on North Atlantic circulation is poorly constrained^33^.

To date, both data and model-based reconstructions of OMZ distributions in the fossil record have been limited due to a paucity of relevant model outputs and empirical constraints. On the modeling side, the inclusion of biological processes within paleoclimate simulations using full-complexity general circulation models is in its infancy. On the data side, there is a dearth of OMZ proxies for water column oxygenation, with most proxies restricted to benthic environments. As a result, past studies have frequently been limited to time periods and locations where the OMZ intersects the seafloor or pelagic anoxia (no oxygen) is extreme enough for indicators of euxinic (anoxic and sulfidic) conditions to be deposited in marine sediments. Some examples of proxy-based OMZ reconstructions include deglacial expansion along continental margins (^15^ and references therein) and the identification of water column euxinia during the Mesozoic Oceanic Anoxic Events^34–36^. However, because OMZs are mid-water phenomena, they are primarily pelagic, rendering them frequently invisible to benthic proxies. Moreover, most modern OMZ waters are not truly anoxic (Fig. 1), much less euxinic. While there are a few additional proxies for pelagic OMZs, such as nitrogen isotopes and organic carbon accumulation, they are responsive to a suite of factors, such as nutrient utilization and preservation frequently making it difficult to tease apart the influence of the various drivers without additional context.

**Fig. 2:**
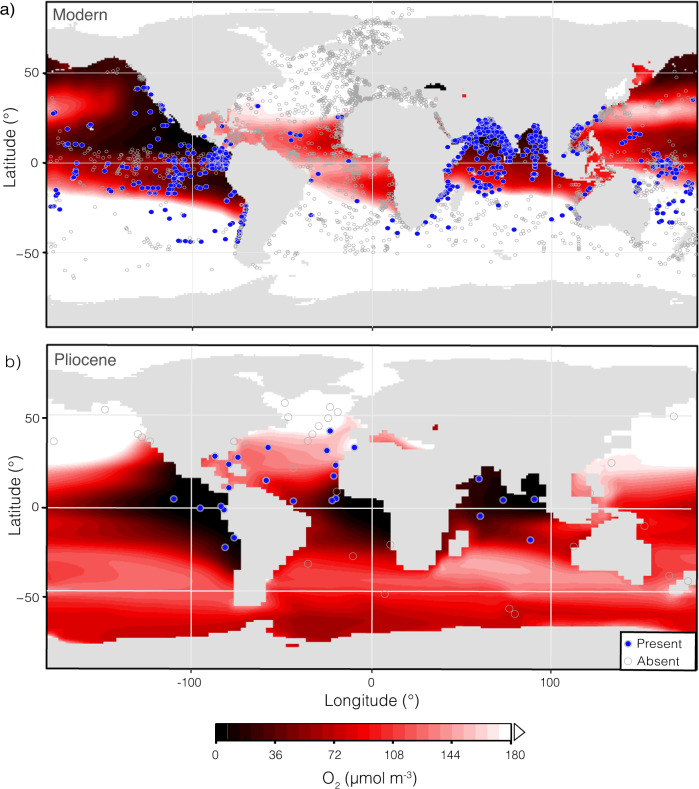
Distribution of low-oxygen affiliated planktic foraminifer *G. hexagonus* and oxygen at ~600 m in the modern and Pliocene oceans. Oxygen concentrations at ~600 m depth **a**) aggregated from WOA18 data (1955-2010)^90^, and **b**) from the Pliocene-like CESM simulation. Sites where *G. hexagonus* was found to be present are shown with blue fill; sites were *G. hexagonus* was absent are open gray points^37–40, 82–87^. Lower O_2_ concentrations are shown in darker red and oxygenation above 172 μmol m^-3^ is white.

Here we present the distribution of *Globorotaloides hexagonus*, a deep-dwelling, OMZ-associated planktic foraminifera^37^, as a proxy for pelagic OMZs. *Globorotaloides hexagonus* has been found at a range of depths (~50–1000 m) and productivity regimes and is consistently associated with sub-thermocline low-oxygen waters (Fig. 2a)^37–40^. By leveraging extensive databases of the distribution of planktic foraminifera in modern and mid-Pliocene oceans^41–43^, Species Distribution Models (SDM), and a biogeochemically enabled global climate simulation resembling Pliocene conditions^25,44,45^, we provide both data-based and model-based constraints on pelagic OMZs in the Pliocene and investigate the dominant drivers of changed OMZ distributions.

## Results & discussion

### Ecological modelling and proxy interpretations of *G. hexagonus*

**Fig. 3:**
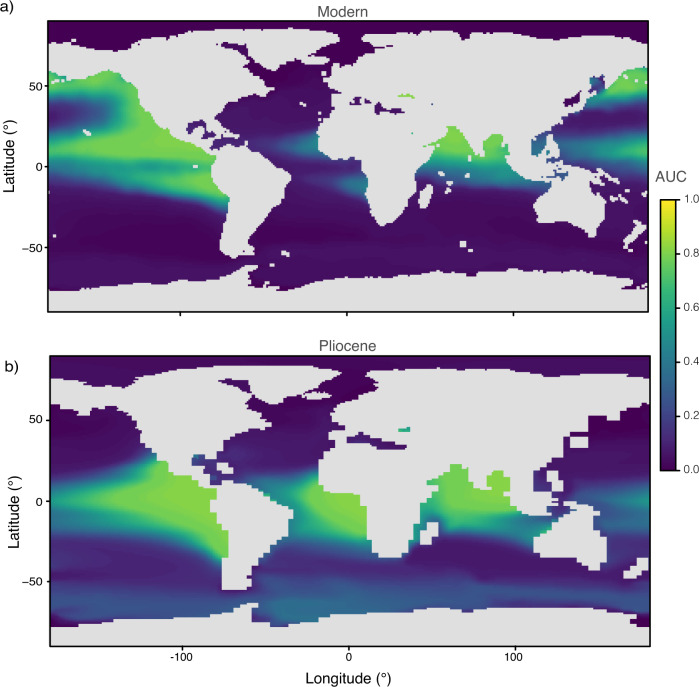
Oxygen-based SDMs showing suitable habitat for *G. hexagonus* in the modern and Pliocene Ocean. Habitat is predicted from **a**) WOA18 oxygen data (1955–2010)^90^ integrated between 400 and 800 m, and **b**) oxygen outputs integrated between 400 and 800 m in the Pliocene-like climate simulation. AUC (true positive rate versus false positive rate) ranges between 0 and 1, with 1 indicating perfect prediction by the model.

Ecological modeling of *G. hexagonus* in the modern ocean supports an association with dissolved oxygen levels at sub-thermocline depths, with results presented here for 400–800 m integrated depth (Fig. 3). Species Distribution Models performed best when using multiple correlated variables (O_2_, temperature, salinity, and macronutrients SiO_3_, PO_4_, and NO_3_). Dissolved oxygen consistently ranked among the most important of these variables, and temperature and salinity among the least important for predicting habitat. After oxygen, silica was the next most important variables at depth followed by phosphate, but both were far less important for models calibrated using surface (0 m) parameters. This effectively demonstrates an affinity of *G. hexagonus* for low oxygen mid-waters, decoupled from surface productivity. We note that the term ‘mid-water’ is used here to describe depths from the thermocline to ~1000 m as distinct from ‘intermediate water’. While the later frequently references specific water masses (e.g., ‘Antarctic Intermediate Water’), both *G. hexagonus* and OMZs may occupy depth ranges not limited to named intermediate water masses.

Species Distribution Models driven by observed ocean conditions confirm the strong relationship between the presence of *G. hexagonus* and low oxygen mid-waters, demonstrating high AUC (0.87; true positive rate versus false positive rate) and percentage correct (84%) prediction scores. Models using dissolved oxygen alone avoid potential overfitting and performed well even compared to those fit with all environmental covariates (Supplementary Table 1). Whether a specific oxygen threshold within the OMZ is required to maintain a population of *G. hexagonus* is currently unresolved. The only published work comparing water-column oxygenation with the depth distribution and abundance of *G. hexagonus* found the species to be most abundant in waters with oxygen below 0.14 ml L^-1^ (~9 µmol m^-3^) in the Eastern Tropical North Pacific, but individuals were found at low densities throughout and just above the oxycline^37^. This may be taken as a guide to the species’ oxygen range, but whether the same oxygen levels predict population abundance and depth distributions globally will require additional sampling.

The preferred oxygen-only SDM indicates almost no viable habitat for *G. hexagonus* in the modern Atlantic Ocean, in line with sparse reported (and disputed), occurrences in the basin (Figs. 2a and 3a) and suggests that the species may be present only as small, restricted populations in the Atlantic. The SDM does predict suitable habitat for *G. hexagonus* in the subpolar North Pacific (Fig. 3a), where it has not been observed in modern samples. It is possible that *G. hexagonus* may be present in this environment but not yet identified, though we think it more likely that their habitat is geographically limited to tropical to transitional zones. Geographic range limits are common for planktic foraminifera, with few truly cosmopolitan species^46^, and our SDM model only accounted for observations (and not absences) given the low detection probability of rare species (e.g., Fig. 2a). Thus, the range of *G. hexagonus* may be bounded by additional geographic or hydrologic constraints, which are not resolved given the modeling approach used. A greater understanding of the ecology and biogeographic limitations of *G. hexagonus* along with alternate modeling approaches will serve to improve future proxy interpretations.

A proxy is definitionally an indirect measure, and paleoceanographic studies are at their strongest when proxy limitations are accounted for and multiproxy or combined proxy-model approaches with different underlying biases and limitations are used. Here we discuss the peculiarities and limitations of the use of *G. hexagonus* as OMZ proxy in terms of “false positives” and “false negatives” given our use of a binary presence/absence metric. For *G. hexagonus*, a false positive can occur through misidentification, lateral transport, or incongruity in timescales of oxygen measurements and species occurrence data (Fig. 2a). For example, sediments typically include a mix of in situ and reworked tests. That means “recent” sediments may contain a few shells from periods during which the configuration of low-oxygen waters may have been different from that in the past half-century. Where *G. hexagonus* tests are found below modern well-oxygenated waters, the species does not exceed 1% of the assemblage (Supplementary Fig. 1). Thus, for a study particularly sensitive to uncertainly arising from false positives, an abundance metric rather than binary presence/absence could be an alternate approach. By contrast, the relative rarity of *G. hexagonus* means that false negatives are probably far more common. In a standard assemblage of 300 individuals, species with a relative abundance <0.3% are likely to go unobserved. This could be a significant source of error for rare species such as *G. hexagonus*, especially at highly productive sites where surface-dwelling foraminifera far outnumber OMZ-affiliated species living at depth. This would be compounded by a bias of human identification away from rare species^47^. For these reasons, the absence of *G. hexagonus* alone in most assemblages, should not be interpreted as a conclusive indicator of a well-oxygenated water column. Absences are only interpreted here in large assemblages (>1800 individuals counted), if replicated across multiple sites, or supported by Community Earth System Model (CESM) outputs.

### Distribution of OMZs in the Pliocene

**Fig. 4:**
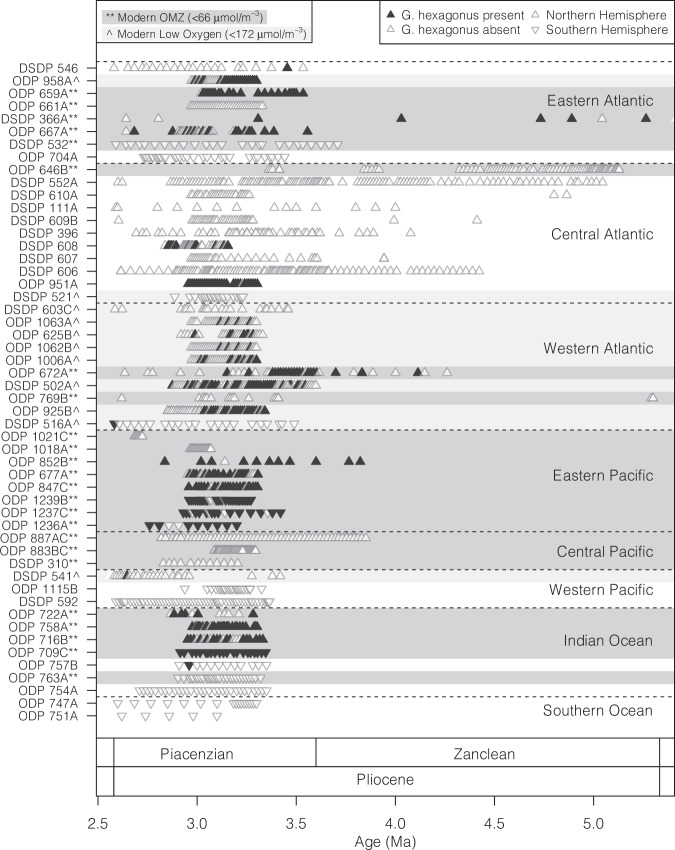
Presence/absence of *G. hexagonus* shells through time. Samples where *G. hexagonus* was present in the PRISM database are shown as filled black triangles; samples where *G. hexagonus* was not found are open gray triangles^41^. Records are organized by region. Sites highlighted by a dark gray box and labeled with a ‘**’ in the y-axis legend are clearly associated with a modern OMZ and oxygen <66 µmol m^−3^. Those with a light gray box and labeled with a ‘^’ in the legend are regions which meet our low-oxygen threshold (<172 μmol m^−3^; Fig. 1) but are not consistently understood to be within permanent modern OMZ. Upward-facing triangles are from sites in the northern hemisphere, while downward-facing triangles indicate sites in the southern hemisphere.

The presence (or absence) of *G. hexagonus* remains largely constant through the Pliocene in deep sea sites from the Pacific, Indian, and Eastern Atlantic oceans (Fig. 4). This is supported by a biogeochemically enabled CESM simulation with an active PMOC and ocean biogeochemistry which predicts low-oxygen mid-waters through all three regions. All reported presences of Pliocene *G. hexagonus* fall within a grid square of low-oxygen mid-waters in the CESM simulation (Fig. 2b; Supplementary Fig. 2). Application of our SDM to the oxygen output from this Pliocene-like CESM simulation indicates suitable habitat for *G. hexagonus* in most low-oxygen mid-waters of the subpolar to tropical regions, with greater suitability around the Equator and in the Southern Hemisphere (Fig. 2b).

The global configuration of Pliocene OMZs inferred from the distribution of *G. hexagonus* and Pliocene-like CESM simulation are broadly similar to modern, with the noteworthy distinction that the Pliocene hosted a greater expanse of low-oxygen waters in the North Atlantic and less in the North Pacific. We note that neither proxy nor model capture high-frequency oceanographic variability on orbital or shorter timescales that could have impacted OMZ distribution over the course of the Pliocene. Thus, the distribution of OMZs described here can be considered a baseline for the distribution of low-oxygen mid-waters given Pliocene climate and circulation parameters.

### Eastern Boundary Currents and evidence for PMOC

Most modern OMZ regions also supported OMZs during the Pliocene. This is despite evidence for less vigorous upwelling-conducive winds and reduced productivity in several key Pliocene EBUSs^28,30–32^. Both the distribution of *G. hexagonus* and the Pliocene-like simulation suggest that at least two EBUSs, the Canary Current System in the eastern North Atlantic and the Humboldt (Peru) Current System in the eastern South Pacific, hosted low-oxygen mid-waters during the Pliocene. This demonstrates the importance of the confluence of low-oxygen source waters with upwelling-driven productivity for fueling OMZs in the Pliocene as in the modern ocean. The CESM output also predicts a low-oxygen region associated with the Benguela Current System in the eastern South Atlantic (Fig. 2b), which our SDM would affirm as suitable habitat for *G. hexagonus* in the Pliocene (Fig. 3b). However, there are no sites in the PRISM database to corroborate the models in this region (Figs. 2b and 3b), with the closest site, DSDP 532, showing *G. hexagonus* absent.

**Fig. 5:**
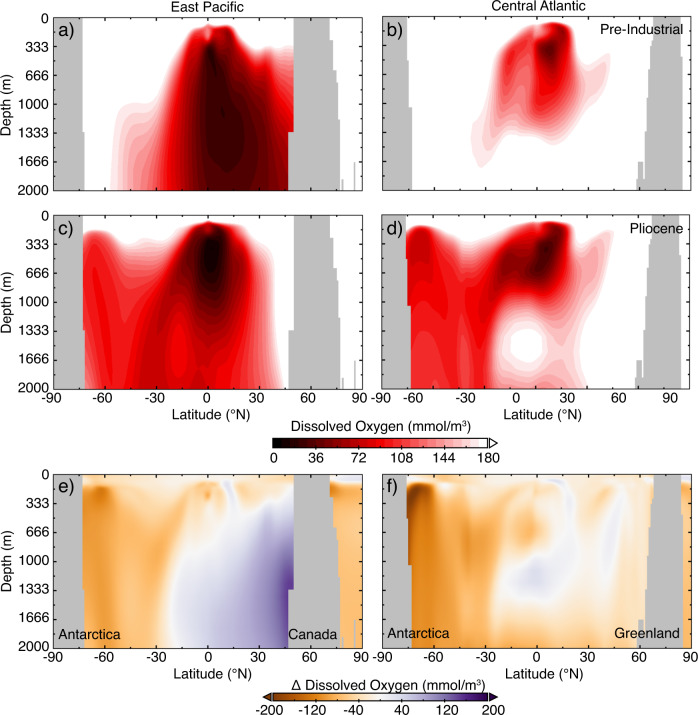
Comparison of oxygen in sections from Pre-Industrial and Pliocene-like simulations. Transects through the East Pacific (130°W) (**a**, **c**, **e**) and Central Atlantic (30°W) (**b**, **d**, **f**) with outputs from the Pre-Industrial control (a, b) and Pliocene-like simulation (**c**, **d**), and the difference in oxygenation between the Pliocene and Pre-Industrial (**e**, **f**) from 0 to 2000 m. Locations of transects are shown in Fig. 1. See Supplementary Fig. 6 to compare modern observational data to the Pre-Industrial control.

While most EBUSs appear to host an OMZ during the Pliocene, *G. hexagonus* is conspicuously absent from Northeast Pacific sites ODP 1021 and 1018 (California Current), both of which underlie a large, established OMZ today. A better oxygenated Northeast Pacific is supported by δ^15^N records from California Margin site ODP 1012, which records relatively little denitrification in the Eastern Tropical North Pacific until ~2.1 Ma^31^. Benthic δ^13^C records from the Southern California margin also indicate more vigorous circulation and increased ventilation^48^. The inference of a relatively well-oxygenated Pliocene California Current System is consistent with the Pliocene-like CESM output due to its simulation of PMOC. Under a PMOC regime both the age and path of intermediate waters circulating through the Pacific would be substantially different from the present day, leading to younger, more oxygen-rich waters being upwelled into the California Current System (Fig. 5).

In the Pliocene-like simulation with PMOC, the Southern Ocean is the endpoint for waters subducted in both the North Pacific and Atlantic. One consequence of this is an increase in Pacific-sourced waters with 100 to 1000 year transit times in the Southern Ocean^49^, and thus an older, more oxygen-depleted Southern hemisphere. Despite low-oxygen mid-waters creating suitable habitat in the Southern Ocean (Fig. 3b), *G. hexagonus* is entirely absent (Fig. 2b). We hypothesize that *G. hexagonus* was excluded from the Southern Ocean due to geographic limitations noted above with regards to the modern subpolar North Pacific. Other alternatives are possible, including that the Pliocene-like simulation predicts a greater degree of oxygen-depletion than was present, but this would need to be tested using alternate proxy approaches.

The Eastern Equatorial Pacific (EEP), associated with the Eastern Pacific cold tongue, is the site of the greatest expanse of low oxygen waters in the modern ocean, driven by both high productivity and low ventilation^17,50^. Our results affirm that an OMZ persisted in this region during the Pliocene. The Pliocene EEP is widely reported to have had a reduced east-west temperature gradient^51,52^, with some authors interpreting warmer EEP temperatures as a decrease in upwelling, implying reduced productivity and thus subsurface respiration compared to today. While some records do show a decrease in EEP productivity centered on the mid-Pliocene^53^, others demonstrate sustained productivity and oxygen-depleted intermediate waters, akin to modern dynamics^54,55^. Warmer Pliocene EEP temperatures could also reflect an altered source of warmer sub-thermocline waters, a hypothesis broadly supported by subsurface temperature proxies^56^, regional pH proxies^57^, and our finding of a low-oxygen water mass occupying EEP mid-waters (Fig. 2).

In addition to the Atlantic and Pacific Ocean EBUS-associated OMZs, there is widespread evidence from sediment geochemistry and benthic foraminiferal assemblages for increased productivity and reducing conditions in the Indian Ocean from the late-Miocene through mid-Pliocene (~6.5-3 Ma), including a potentially expanded OMZ relative to the Pleistocene^58–61^. A strong, Indian Ocean OMZ is supported by the presence of *G. hexagonus* in the northern Indian Ocean during the Pliocene and the modeled extent of low oxygen conditions (Fig. 2b), affirming previous work. This result is consistent with an influence of low-oxygen Southern Ocean-sourced waters during the Pliocene.

### North Atlantic Pliocene OMZs

One striking difference between modern and Pliocene distributions of low-oxygen waters is in the western North Atlantic, including the Caribbean Sea and portions of the Subtropical Gyre. Both are regions with relatively high subsurface oxygen in the modern ocean but for which there is both proxy and model support for low-oxygen waters during the Pliocene. For much of the western North Atlantic, altered intermediate water circulation is the likely source of decreased oxygenation. These regions are currently ventilated by AAIW^62^. However this arrangement was proceeded by a warmer, saltier, Pacific-sourced ‘proto-AAIW’ prior to ~ 3 Ma^63^ with lower O_2_ content. The presence of a poorly oxygenated proto-AAIW would have influenced the Canary Current System in the eastern North Atlantic, where modern AAIW is a significant contributor to OMZ depth and deeper (>600 m) waters^64^. The potential for OMZ formation in the North Atlantic is particularly salient given observations of warming and deoxygenating source waters for these regions over the past decades^11^.

There is substantial evidence for both high productivity and the presence of Western Atlantic OMZs in warm climates preceding the Pliocene, with benthic foraminiferal assemblages reflecting a well-developed OMZ in the western Caribbean in the late Miocene^65,66^. While little direct evidence exists for a benthic OMZ persisting into the Pliocene, paleontological and geochemical records suggest a more productive Western Atlantic in the Pliocene relative to the Pleistocene. Ichthyofaunas reflect an upwelling ecosystem around the Cariaco trench^67^, while ostracod assemblages indicate cooler, upwelling-associated temperatures along the Southwest Atlantic margin^68^. In the eastern Gulf of Mexico, high mid-Pliocene productivity is associated with stronger-than-modern upwelling^69^. Similarly, upwelling-associated planktic foraminifers *Neogloboquadrina pachyderma* and *Globigerina bulloides* are abundant in the Western Caribbean in the Late Pliocene^70,71^. Trace elements, as well as CaCO_3_ and SiO_2_ mass accumulation rates from ODP Site 999 suggest heightened export productivity until ~1.7 Ma^72^. At Ceara Rise ODP Site 925, elevated productivity compared to present day persisted until at least ~3.7 Ma^73^, while mid-Pliocene (~4-3 Ma) δ^13^C of thermocline dwelling *Neogloboquadrina dutertrei* is lower than either surface-dwelling *Trilobatus sacculifer* or Holocene *N. dutertrei*^74^. Together, these lines of evidence all indicate elevated Pliocene upwelling-driven productivity, consistent with the presence of an OMZ.

A shift from the presence to a consistent absence of *G. hexagonus* tests in several North Atlantic sites could suggest a regional change occurring during the mid-Piacenzian. The species apparently disappears from Northwest Atlantic ODP Sites 1063, 625, 672, and 925 in the Western Atlantic between 3.5 and 3 Ma, as well as from ODP Site 958 in the Eastern Atlantic (Fig. 4). While precise dating of this apparent shift is limited by data availability, the timing would be consistent with either an evolution of source-waters from a Pacific-influenced proto-AAIW to a more modern AAIW composition^63^, or reduced upwelling and decreased productivity in the marginal Western Atlantic seas.

High productivity in the early and mid-Pliocene Western Atlantic has traditionally been attributed to the inflow of more nutrient-rich waters across the Central American Seaway^75^. The aftermath of seaway closure is linked with the development of the Western Atlantic Warm Pool^76^, which decreased productivity and facilitated the expansion of reef ecosystems throughout the Caribbean^77,78^. The timing of Central American Seaway closure remains debated^79^. Geochemical differentiation of surface waters likely began as early as ~4.2 Ma^80^ with the development of a warm pool by 4 Ma^76^, and an increasingly oligotrophic Caribbean emerging between 4.2-2.5 Ma^81^. However, other authors show potentially intermittent surface connectivity persisted until as recently as 3-2.5 Ma^79^. Notably, the CESM used here is parametrized with a fully formed Panama Isthmus. Thus, while the Central American Seaway may have contributed to increased productivity and remineralization in the Pliocene Western Atlantic, it appears not to be a necessary condition for circulatory changes that may produce a Western Atlantic OMZ (Figs. 2, 5). This finding has particular significance for the future ocean, as Southern Ocean-sourced intermediate waters in the Atlantic have been deoxygenating since the mid 20^th^ century^11^. Thus, the Pliocene may offer a meaningful reference point, demonstrating the potential for Atlantic OMZ expansion, with a modern continental configuration.

Warm, poorly ventilated mid-waters allowed for widespread OMZs in the Pliocene as evidenced by the distribution of the low-oxygen affiliated species *G. hexagonus* and supported by both Earth System and Species Distribution Models. This includes maintenance of low-oxygen waters underlying the Humboldt Current System in the eastern South Pacific and the northern Indian Ocean similar to modern day. Low-oxygen mid-waters were also pervasive in the North Atlantic through the mid-Piacenzian, reflecting an evolution from a proto-AAIW water mass to a modern, better oxygenated intermediate water configuration in the Late Pliocene. In contrast to the modern ocean, the Pliocene California Current EBUS appeared well-oxygenated, consistent with altered intermediate water circulation associated with a Pliocene PMOC. Thus, a warmer world can support both regionally expanded and contracted OMZs, influenced by distant intermediate water formation and circulation. The close ties between *G. hexagonus* and modern OMZs and the striking data-model match found here, suggest that the species has enormous potential to reveal the presence and evolution of pelagic low-oxygen water masses.

## Methods

There is no single definition of an OMZ, with quantitative thresholds varying between contexts and studies. Here, we account for regions where waters shallower that 1000 m reach oxygen concentrations of 172 μmol m^-3^ or less (Fig. 1). This value has been chosen to be inclusive of all major regions of the modern ocean that are frequently described as possessing an OMZ and to account for differences in OMZ depth (Supplementary Fig. 2). Unless otherwise stated, O_2_ maps were made with the use of NASA’s “Earth” overlay (https://www.giss.nasa.gov/tools/panoply/).

### Presence of *G. hexagonus*

The distribution of *G. hexagonus* tests in marine sediments is used as an indicator of overlying oxygen-depleted mid-waters in the Pliocene. We interpret data only in terms of presence/absence. This has been done in part to account for unconstrained differences in sedimentation rate and preservation between sites. Moreover, modern observations show that *G. hexagonus* is likely not sharing a habitat with the planktic foraminifera species abundant above the oxycline^37^. Due to this fundamental difference in habitat and very low densities of *G. hexagonus* in the modern ocean^37^, metrics such as relative abundance will be at least as responsive to varying abundances of shallow-dwelling species as mid-water species such as *G. hexagonus*.

The presence or absence of *G. hexagonus* in the Pliocene was assessed based on a database of planktic foraminifera faunas published as a part of the PRISM project^41^. The PRISM project focuses on the mid-Piacenzian Warm Period (~3.3-3.0 Ma), with the largest density of samples clustered around this interval^41^, but contains samples dated from 6.2 to 1.8 Ma. Only data from the Pliocene (5.3-2.6 Ma) is included here (Fig. 4). A modern comparison draws on faunas from the Brown University Database (BFD) and ForCenS Database of recent sediments^42,43^, and reports from plankton tows and sediment traps^37–40,82–87^ (Supplementary Figs. 1, 3). Given the relative rarity of *G. hexagonus* in both modern and Pliocene sediments (<3%), absences are considered meaningful only if counts at a site are sufficient to capture a relative abundance of at least 0.1%. One outcome of this threshold is that absences from “modern” sites (~300 count/site in ForCENs) are not formally considered, while they are from the PRISM database (> 1800 count/site).

### Ecological modeling of *G. hexagonus*

The distribution of *G. hexagonus* in the modern ocean along with physical and biogeochemical environmental conditions and a variety of statistical/machine learning algorithms was used to estimate *G. hexagonus* habitat suitability in the modern ocean. This ecological model was then driven by corresponding environmental variables from a simulated Pliocene-like ocean (see next section) to project habitat suitability under Pliocene conditions. Data for *G. hexagonus* occurrence in the modern ocean was taken from the BFD (Atlantic only) and ForCenS (other basins) Databases^42,43^ and converted to presence/absence (Fig. 2a; Supplementary Fig. 3). The use of the BFD in the Atlantic was chosen so as not to presuppose the absence of *G. hexagonus* from this basin in our models.

Modern environmental covariate data, including temperature^88^, salinity^89^, dissolved oxygen^90^ nitrate, phosphate, and silica^91^ were taken from WOA18 at depths ranging from 0–1000 m. SDMs were constructed from the relationship between occurrence (presence only) and environmental covariate data using a variety of statistical (logistic regression, General Additive Models) and machine learning (Boosted Regression Trees, Random Forests, Maximum Entropy) algorithms commonly employed in SDMs^92^. Each model was run multiple times with 5-fold cross validation to assess performance and robustness. Models using both all variables and dissolved oxygen only were constructed and compared.

### Physical and biogeochemical modeling of Pliocene conditions

This study makes use of Pre-Industrial and Early (~4–5 Ma) Pliocene-like climate simulations performed using the coupled ocean-atmosphere Community Earth System Model (CESM) version 1.2.2. While a detailed description of these simulations is provided elsewhere^57^, we will briefly summarize here. These simulations have active ocean biogeochemistry^93^ and use the T31 gx3v7 configuration designed for long paleoclimate simulations^94^^.^ In our case these have been run for 3000 years, allowing the ocean to reach quasi-equilibrium^57^ (Supplementary Fig. 3). The atmospheric (Community Atmosphere Model 4) and land surface components (Community Land Model 4) have a spectral truncation of T31, and the oceanic (Parallel Ocean Program 2) and sea ice components (Community Ice Code) have a resolution ranging from 3° near the poles to 1° at the equator. The design for the Pre-Industrial experiment consists simply of the default B_1850_BGC-BDRD CESM component set (Supplementary Fig. 4). For the Pliocene experiment, cloud albedo is reduced in the extratropics and increased in the tropics by modifying the liquid and ice water path in the shortwave radiation scheme following the design of^25,44,45^. All other boundary conditions and radiative forcings in the Pliocene experiment are the same as the Pre-Industrial control. The modifications to cloud shortwave radiative forcing lead to large-scale warming patterns that largely reproduce the meridional and zonal gradients seen in Pliocene SST reconstructions^45,95^. These ocean warming patterns in turn influence the large-scale hydrological cycle^28^ which has important implications for the density of surface waters in the subpolar North Pacific leading to deep water formation and a PMOC, which influences oxygen^25,57^ (Supplementary Figs. 5, 6). The analysis presented here is based on the last 100 years of each simulation (years 2901–3000).

## Supplementary information

Supplementary Information

## Data Availability

All data associated with this manuscript have been previously published and is freely available as part of the PRISM (https://www.ncei.noaa.gov/access/paleo-search/study/19281), ForCenS (10.1594/PANGAEA.873570), and Brown Foraminiferal Databases (10.1594/PANGAEA.96900). Model outputs are available at XXXX.

## Code availability

Climate simulation and habitat suitability output data for this study will be uploaded to Zenodo (https://zenodo.org/deposit/7254850).
