# Supplementary material for: Intermediate water circulation drives distribution of Pliocene Oxygen Minimum Zones
Source: Nat Commun. 2023 Jan 4;14:40. doi: 10.1038/s41467-022-35083-x (PMC9812982; doi:10.1038/s41467-022-35083-x)
Supplement: Supplementary file 1 — Supplementary Information [file 41467_2022_35083_MOESM1_ESM.docx]

**Supplement to Intermediate water circulation drives distribution of Pliocene Oxygen Minimum Zones**

Catherine V. Davis^1,*^, Elizabeth C. Sibert^2,3^, Peter H. Jacobs^4,5^, Natalie Burls^6^, Pincelli M. Hull^2,7^

^1^Department of Marine, Earth, and Atmospheric Sciences, North Carolina State University, Raleigh, NC, USA

^2^ Department of Earth and Planetary Sciences, Yale University, New Haven, CT, USA

^3^ Yale Institute for Biospheric Studies, Yale University, New Haven, CT USA

^4^ Department of Environmental Science and Policy, George Mason University, Fairfax, VA, USA

^5^ Currently: Earth Science Division, NASA Goddard Space Flight Center, Greenbelt, MD, USA

^6^ Department of Atmospheric, Ocean & Earth Sciences, George Mason University, Fairfax, VA, USA

^7^ Yale Peabody Museum of Natural History, Yale University, New Haven, CT, USA

^*^ catherinedavis@ncsu.edu

| **Information** | **Oxygen Only** | **Multi-variate** | **Description(s)** |
| --- | --- | --- | --- |
| Variables | O_2_ | O_2_, SiO_3_, PO_4_, T, S, NO_3_ | Environmental covariates used in model |
| Depth | 400 to 800m | 400 to 800m | Depth of environmental covariate(s) |
| Seed | 5000 | 5000 | Random seed |
| AUC | 0.87 | 0.92 | True Positive (TP) rate vs. False Positive (FP) rate (Sensitivity vs. 1 - Specificity) |
| Kappa | 0.56 | 0.68 | Observed accuracy vs. expected accuracy due to chance |
| Omissions | 0.42 | 0.34 | Percent incorrect absence predicted (False Negative (FN) / (FN + True Negative (TN))) |
| Sensitivity | 0.58 | 0.66 | Percent correct presence predicted (TP / (TP + FN)) |
| Specificity | 0.94 | 0.97 | Percent correct absence predicted (TN / (TN + FP)) |
| Percent Correct | 0.84 | 0.88 | Correct predictions vs. total samples |

**Supplementary Table 1.** Selected MaxNet models are shown. The dissolved oxygen only model is compared to a model fit using a full range of World Ocean Atlas variables with performance statistics and their descriptions. Including more variables generally increases model performance statistics at the risk of overfitting. Here the oxygen-only model performs comparably to the full-variable model (e.g. 0.87 vs. 0.92 AUC). SDMs were run using a variety of common statistical and machine learning algorithms, including Logistic Regression, Gradient Boosting, MaxEnt, MaxNet (an open-source reimplementation of MaxEnt), and Random Forests. Each model was rerun multiple times using randomly withheld data for cross-validation (k = 5). While Random Forest models slightly outperformed the MaxNet models, this came at the expense of overfitting as evident in unrealistically discrete spatial patterns.


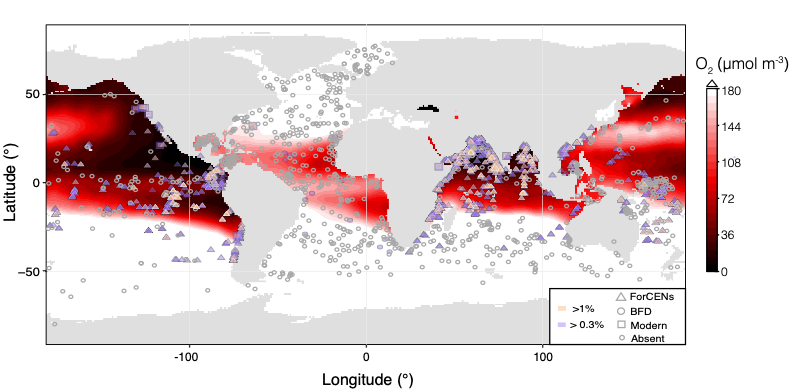


**Supplementary Figure 1.** Modern oxygen minimum distributions based on aggregated WOA18 oxygen data collected in years 1955-2010^1^, with lower O_2_ concentrations shown in darker red. Abundances of *G. hexagonus* > 0.3% of the assemblage are shown in purple, and > 1% in pink. Absences are shown as open grey circles. Shapes indicate different sources: ForCenS (triangles)^2^, the Brown Foraminiferal Database (Atlantic Basin only; circles) and sediment trap and plankton tow data (squares)^3-11^. Figure is meant to be illustrative of the potential to use a threshold such as >1% (or at least 3 individuals) to avoid ‘false positives’. However, small differences in relative abundance are not likely representative of differing environments.

**Supplementary Figure 2.** Oxygen at depth from WOA18. Each panel integrates over ~25-50 m with the midpoint labeled above the panel.

Supplementary Figure 3. Modern *G. hexagonus* distribution by data source. The presence (solid blue circles) and absence (open gray circles) of *G. hexagonus* from ForCENs (top), the Brown Foraminiferal Database (Atlantic Basin only; middle) and published sediment trap and plankton tow data (bottom). Base map made with Natural Earth (http://www.naturalearthdata.com).

**Supplementary Figure 4.** Comparison of oxygen at ~600 m between (A) modern aggregated WOA18 data (1950-2018) ^1^ and (B) dissolved oxygen output from the Pre-Industrial CESM simulation. Lower O_2_ concentrations are shown in darker red

**Supplementary Figure 5.** Oxygen at depth through the Pliocene-like simulation. Slices are chosen to best compare to Supplemental Figure 2 and the layer nearest to each slice is shown.

**Supplementary Figure 6.** Transects through the East Pacific (130°W) (A, C) and Central Atlantic (30°W) (B, D) show modern WOA18 data (1955-2010)^1^ and (A, B), outputs from the Pliocene simulation (C, D).

**Supplementary Figure 7.** Minimum O_2_ within the upper 1000 m from the Pliocene-like CESM simulation is shown with lower O_2_ concentrations are shown in darker red. Regions where oxygen concentrations do not fall below 172 μmol m^-3^ are white.

**Supplementary References**

1 Garcia, H. *et al.* World Ocean Atlas 2018, Volume 3: Dissolved Oxygen, Apparent Oxygen Utilization, and Dissolved Oxygen Saturation (2019).

2 Siccha, M. & Kucera, M. ForCenS, a curated database of planktonic foraminifera census counts in marine surface sediment samples. *Scientific Data* **4**, 170109, doi:10.1038/sdata.2017.109 (2017).

3 Birch, H., Coxall, H. K., Pearson, P. N., Kroon, D. & O'Regan, M. Planktonic foraminifera stable isotopes and water column structure: Disentangling ecological signals. *Marine Micropaleontology* **101**, 127-145 (2013).

4 Davis, C. V., Hill, T. M., Russell, A. D., Gaylord, B. & Jahncke, J. Seasonality in planktic foraminifera of the central California coastal upwelling region. *Biogeosciences* **13**, 5139 (2016).

5 Davis, C. V., Wishner, K., Renema, W. & Hull, P. M. Vertical distribution of planktic foraminifera through an oxygen minimum zone: how assemblages and test morphology reflect oxygen concentrations. *Biogeosciences* **18**, 977-992 (2021).

6 Fairbanks, R. G., Sverdlove, M., Free, R., Wiebe, P. H. & Bé, A. W. Vertical distribution and isotopic fractionation of living planktonic foraminifera from the Panama Basin. *Nature* **298**, 841-844 (1982).

7 Marchant, M., Hebbeln, D. & Wefer, G. Seasonal flux patterns of planktic foraminifera in the Peru–Chile current. *Deep Sea Research Part I: Oceanographic Research Papers* **45**, 1161-1185, doi:10.1016/S0967-0637(98)00009-0 (1998).

8 Ortiz, J. D., Mix, A., Rugh, W., Watkins, J. & Collier, R. Deep-dwelling planktonic foraminifera of the northeastern Pacific Ocean reveal environmental control of oxygen and carbon isotopic disequilibria. *Geochimica et Cosmochimica Acta* **60**, 4509-4523 (1996).

9 Rao, K. K., Jayalakshmy, K., Kumaran, S., Balasubramanian, T. & Kutty, M. K. Planktonic foraminifera in waters off the Coromandel coast, Bay of Bengal. (1989).

10 Rippert, N. *et al.* Constraining foraminiferal calcification depths in the western Pacific warm pool. *Marine Micropaleontology* **128**, 14-27 (2016).

11 Smart, S. M. *et al.* Ground-truthing the planktic foraminifer-bound nitrogen isotope paleo-proxy in the Sargasso Sea. *Geochimica et Cosmochimica Acta* **235**, 463-482 (2018).
